# Soldier Health

**Published:** 1861-07

**Authors:** 


### "SOLDIER HEALTH."

By Dr. W. W. Hall, 42 Irving Place, New-York. Sent post-paid for 25 cents. 128 pp., 16mo. How to guard against the three prevalent diseases in all armies, Fever, Diarrhea, and Dysentery; and also to control them by means which the soldier may almost any where command; a complete system of camp-cookery and hospital diet, with Scripture-reading and Hymn for each day, with a night, morning, and Sunday prayer and hymn; radiating distances from Washington, Baltimore, Harper's Ferry, Cairo; cost of all the forts; census of all the States—of the militia of each State; fifty-nine health axioms; rank and pay of all the officers and privates in the army, etc., etc. This is a book which would benefit every soldier physically, mentally, and morally.

We conceive it impossible for any reader to spend twenty-five cents to greater advantage than by ordering one of these books to be sent to some friend or kinsman in the army. It is six inches by four, a quarter of an inch thick, has a paper-cover, and weighs less than three ounces, hence can easily be carried to the battle-field in the pocket. It is the only book published that meets so many of the wants of the soldier on the field of battle. If he is left for dead, or wounded, its directions meet the emergencies; if life is ebbing away, there is Scripture-reading adapted to his case, and near a dozen of the sweetest hymns in the English language; hymns as familiar to every American soldier as the alphabet, with their hallowed associations of home and the village-church. When it is remembered that soldiers are not allowed to carry any thing into battle but the indispensable clothing, the arms and the canteen, that often not only the pockets are filled with cartridges, but even the spaces between the buttonings of their coats, a little book of this sort, which can be carried in a very small space, answering to the general wants of the soldier, as to the body and the soul, is of the utmost importance. But all do not think as well of the book as we do. The Philadelphia *Press* of June 17th says:

"Nearly forty pages here, containing Scriptural selections and hymns, seem to have been introduced merely to swell up the work. Dr. Hall's hymns abound in bad rhymes. He makes *cross* rhyme with *cause*; *flood* with *God*; *surpass* with *grace*; *clean* with *sin*; *confess* with *grace*; *song* with *tongue*; *holiness* with *peace*; *been* with *sin*; *power* with *more*; *bleed* with *head*; *done* with *unknown*; *face* with *thankfulness*; *owe* with *do*; *grace* with *praise*; *sin* with *clean*; *past* with *rest*; *shed* with *plead*, and so on. We have seldom found so many miserable rhymes in such a small space. Dr Hall evidently has no ear for rhythm and rhyme."

As Isaac Watts wrote nearly all the hymns, and they are those which have been most admired for a century by nearly all denominations of Christians, we have merely sided with the majority, and rather think that the "Cricket" is not a very regular church-goer. The first lines of the hymns are:

Am I a soldier of the cross?
Alas! and did my Saviour bleed?
God bless our native land.
It is the Lord enthroned in light.
I love to steal awhile away.
Jesus, my all, to heaven is gone.
Jerusalem, my happy home.
My soul, be on thy guard.
My country, 'tis of thee.
Oh! for a thousand tongues to sing.
O thou! who driest the mourner's tears.
Once more, my soul, the rising day.
Show pity, Lord! O Lord! forgive.
The day is past and gone.
There is a fountain filled with blood.
There is a land of pure delight.
Whilst thee I seek, protecting power.
When all thy mercies, O my God!
When I can read my title clear.
Where will be the birds that sing, a hundred years to come?
Ye sons of freedom, wake to glory, (the Marseillaise Hymn.)

It should be remembered that almost all the soldiers are the children of parents, who accustomed them to meet in public worship on the Sabbath-day; very many of them are members of the church and Christian men, and to all these, the memories associated with those dear familiar hymns are inexpressibly dear when away from home, when suffering in an hospital, or among the wounded on the battle-field. It was for this reason they were selected, and not merely "to fill up the book." This world is not the last of man. The preservation of his mortal body is not his chief concern. There is something of immeasurably greater importance than saving life. For "what shall a man be profited if he shall gain the whole world, and lose his own soul?" and to be hurried from the fierce fight into the presence of the Infinite, or to languish in a dreary hospital, for hours, and days, and weeks, and to have no verse or line to help the laboring mind to thoughts of its Maker, is fearful to think of; hence our little volume, which is literally the soldier's "*vade mecum*," a thing which he may well command to go with him wherever he goes himself. It is such a book as the volunteers, who have been brought up in Christian homes, have a right to demand of the Government; and in the language of the Boston *Atlas* and *Bee*, "fifty thousand ought to be sent at once to Washington." We took occasion to say that Christian men were the most reliable patriots, and made the bravest soldiers, the most enduring; for such specially we wrote the book, and they will prize it most. A great deal of most valuable information is given, which does not pertain to health or religion, and this was put in, in order to make those take care of the book, for the sake of this, who would not have done so, merely for the matters of health, morals, or religion. We say, distinctly, that thousands of lives would be saved, and millions of money, within the present year, if the suggestions of the book were followed, in reference to early breakfast, camp-fires, and the practicable prevention of the three great diseases of armies, to wit: fever, diarrhea, and dysentery: their prevention and their cure, in most cases by means which the poorest soldier in the most out-of-the-way place can command; and such is the characteristic of most of the directions given as to health and wounds. The book was not written in the presumption that the soldier was in a drug-store; or in the midst of a large city. Lint will staunch a bleeding wound, but lint may not be had when gunpowder and spider's-web may. A tourniquet may stop a bleeding artery, where death would follow in ten minutes, or less, if not arrested; but tourniquets don't grow in the woods, or out of a sand-bank; but every soldier can command a stick or ramrod, and a handkerchief or a strip of shirt, and without a surgeon or a tourniquet, he can save his life himself. Hartshorn will cure almost every poisonous sting or bite, but hartshorn does not bubble up at every spring, nor flow beside every river, but it is an alkali, and so is wood-ashes, made into a poultice with water, saliva, or a man's own blood, and where there is a stick of wood and a flint or a percussion-cap, wood-ashes can be made. A man may be attacked with wasting diarrhea, or threatened with cholera; he may not have any blackberry cordial at hand, or Dover's powder, or sugar of lead, but he has a trowser-leg or a flannel shirt, and he can lie down in perfect quietude, and these are a thousand times more certain of cure, and a thousand times more safe, and a thousand times less hurtful, than the "authorized" remedies named, as whole armies have found more than once before to-day. So that the commendation which the *American Presbyterian*, of Philadelphia, has given of "Soldier Health," is timely, just, and judicious.

"Dr. W. W. Hall, of New-York, has done good service for our soldiers in his little work on Soldier Health, which is full of direct, intelligible, and forcible hints to soldiers, by an experienced writer on such topics. It will be found greatly serviceable both in preventing and remedying disease, accident, and dissipation in this class of men, and it is written in a style which will not fail to command their attention. The book is indeed quite a *vade mecum*, containing religious reading, hymns, prayers, Soyer's Army Receipts, and selected information on military and other matters which soldiers especially would be interested in knowing."

The main, in fact, the only objection that Andrew Jackson Davis, editor of the *Pilgrim's Progress*, makes to the book is, that there is a foot-note to one of the three prayers. Now, friend Andrew, we didn't intend the note to be prayed; it was only to show the soldier how much our Revolutionary sires endured when they fought for liberty, in order to encourage the volunteers of to-day to "grin and bear" bravely any little uncomfortablenesses which they might be called to encounter, such as having to dig without gloves, to march without parasols, or take their dinners without a four-pronged silver fork. So we think our "foot-note" was very much to the point. To show the correctness, and also the importance of our statement, that fevers, diarrhea, and dysentery were the chief diseases of camp life, we give an official statement that, of the nine hundred and ninety-seven hospital cases at Cairo, in the two weeks ending with June 7th, 1861, seven hundred and twenty-seven were of those three diseases, nearly every one of which was certainly preventable without one dollar's extra expense, only with a little knowledge and a little attention. To give that knowledge, and to show how to pay that attention, is one of the great aims of the book, which will be furnished to individuals and societies, for gratuitous distribution among the soldiers, at a considerable per centage below half the retail price.

As an evidence of the felt importance of our suggestions in reference to the preservation of the health of the soldiers, every book but one that we have seen of that kind, and there are several, copies our own, *verbatim, et literatim, et punctuatim*, errors and all.

